# Sex and gender differences in treatment intention, quality of life and performance status in the first 100 patients with periampullary cancer enrolled in the CHAMP study

**DOI:** 10.1186/s12885-023-10720-w

**Published:** 2023-04-11

**Authors:** Sofie Olsson Hau, Caroline Williamsson, Bodil Andersson, Jakob Eberhard, Karin Jirström

**Affiliations:** 1 Division of Oncology and Therapeutic Pathology, Department of Clinical Sciences, Lund University, SE-221 85 Lund, Sweden; 2 Division of Surgery, Department of Clinical Sciences, Lund University, Lund, Sweden

**Keywords:** Periampullary cancer, Pancreatic cancer, Chemotherapy, Sex, Gender, Quality of life, Performance status, Treatment intention

## Abstract

**Background:**

Periampullary cancer is a term for cancers arising in or in close proximity to the pancreas. Pancreatic cancer is the 3^rd^ leading cause of cancer death for both sexes and while surgery is the only option for cure, chemotherapy is given in both the adjuvant and palliative settings. The aim of this study was to investigate any sex and gender differences in patients with pancreatic and other periampullary adenocarcinomas enrolled in a prospective, observational trial.

**Methods:**

The study cohort consists of the first 100 patients, 49 women and 51 men, enrolled in the Chemotherapy, Host Response and Molecular dynamics in Periampullary cancer (CHAMP) study, an ongoing study of patients undergoing neoadjuvant, adjuvant or first-line palliative chemotherapy treatment. Twenty-five patients had surgery with curative intent and subsequent adjuvant treatment, and 75 patients were treated with palliative chemotherapy. Data regarding health-related quality of life (HRQoL, EORTC-QLQ-C30) at baseline, demographic and clinicopathological factors were examined and stratification by treatment intention according to sex. Overall survival (OS) was calculated through Kaplan–Meier analysis.

**Results:**

There was a statistically significant difference between male and female patients treated with curative intent, with fewer women having undergone surgery (18 vs 7, *p* = 0.017), also after adjustment for age, tumor location and performance status. No statistical differences were found between the sexes regarding age, comorbidities, or clinicopathological factors. Before start of chemotherapy treatment, health-related quality of life (HRQoL) was lower in female than in male patients. However, HRQoL was not associated with performance status in female patients, whereas in male patients several HRQoL indicators were significantly positively associated with poorer performance status at baseline.

**Conclusions:**

This study shows no clear differences between the sexes regarding biological factors concluding that gender bias might be responsible for the discrepancy between men and women being offered curative surgery. The observed difference between women and men regarding the association between HRQoL and performance status is unprecedented. Altogether these findings underline the importance of taking gender into consideration when assessing eligibility for curative surgery in order to improve biological outcome and decrease suffering in both sexes.

**Trial registration:**

NCT03724994.

**Supplementary Information:**

The online version contains supplementary material available at 10.1186/s12885-023-10720-w.

## Background

The term periampullary cancer encompasses a heterogenous group of tumors arising in the pancreas, the Ampulla of Vater, distal bile duct or the periampullary duodenum. The majority of periampullary tumors are located within the pancreas. These tumors carry a dismal prognosis and afflicted patients often suffer from severe symptoms. In the United States it is estimated that pancreatic cancer will be responsible for 8% of cancer deaths in 2022. At present, it is the third leading cause of cancer death globally but is projected to rise to the second by the year 2030. Pancreatic cancer incidence is slightly higher in men than in women, with an estimated 53% of newly diagnosed patients being male [1, 2]. Biological sex modulates cancer through genetic and hormonal influences while the social constructs of gender affect the way patients and the health care system interact. Although a growing field, the influence of sex and gender on cancer evolution and response to treatment has hitherto been understudied [3]. As in many other types of cancer, men with pancreatic cancer have decreased survival in late-stage disease compared to women [4, 5]. Lifestyle factors such as smoking, excessive alcohol consumption and an unhealthy diet are more common in men, and their poorer survival has often been attributed to these factors. However, one study of pancreatic cancer conducted using a mouse model shows a clear sex difference in survival with decreased survival and increased liver metastases for male mice without exposure to extrinsic factors, further supporting that lifestyle factors alone cannot explain the differences in pancreatic cancer survival between the sexes [6].

The majority of patients with periampullary cancer present with irresectable disease, and palliative chemotherapy remains standard of care, albeit leading to only modest survival benefits. Patients with operable disease undergo surgery followed by adjuvant chemotherapy, however, most relapse and the 5-year overall survival is 5–28% globally [7]. In a recent Swedish registry study, female patients who were planned for curative pancreatic cancer surgery had significantly better long-term survival than male patients, although they were older at diagnosis (69 vs 68 years of age) [8].

Chemotherapy is given in both adjuvant and palliative settings as well as for downsizing borderline resectable tumors [9]. Combination treatment with FOLFIRINOX (folinic acid, 5-fluorouracil, irinotecan, and oxaliplatin) or modified FOLFIRINOX is standard of care for fit patients, while patients with poorer performance status receive single agent chemotherapy with gemcitabine or 5-FU (Fluorouracil). For patients who are deemed unfit for FOLFIRINOX, but well enough to tolerate combination chemotherapy, gemcitabine in combination with nab-paclitaxel or capecitabin is given [9–18]. Many patients suffer from toxicity to treatment, and even with optimal combination chemotherapy treatment the overall survival for stage IV patients is less than a year [12, 19]. Female patients are subject to a 1.5 times higher risk of serious drug side effects compared to males and in women with pancreatic cancer there is an increased risk of toxicity from 5-FU based chemotherapy [20, 21].

The aim of this study was to investigate any differences between males and females regarding demographic and clinicopathological parameters as well as treatment intention, performance status, quality of life and overall survival (OS) in the first 100 patients enrolled in the Chemotherapy, Host Response and Molecular dynamics in Periampullary cancer (CHAMP) study [22].

## Methods

The CHAMP study is an ongoing prospective, single-arm observational study registered in clinicaltrials.gov as NCT03724994 [22]. All patients with a histologically or cytologically confirmed diagnosis of pancreatic or other periampullary adenocarcinoma undergoing adjuvant or palliative chemotherapy treatment in the Department of Oncology, Skåne University Hospital have been invited to participate, and the first patient was included in November 2018.

Clinical and pathology data are compiled at study entry with radiological and clinical follow-up being performed at three-month intervals. Serial blood sampling during chemotherapy treatment is performed by a dedicated research nurse and health-related quality of life (HRQoL) is assessed every three months through EORTC-QLQ-C30 (The European Organisation for Research and Treatment of Cancer Quality of life Questionnaire) [23]. In May 2022, 100 patients had been included, of whom 49 participants were female and 51 male. The latest follow-up was on 31^tst^ October 2022. Seventy-five patients had completed EORCT-QLQ-30 questionnaires at baseline, i.e. before the first chemotherapy cycle (6–8 weeks post-operatively for adjuvant patients). Comorbidities and risk factors were established through examination of patient charts. Cardiac comorbidity was defined as patients having a history of myocardial infarction, frequent angina pectoris or heart failure. Atrial fibrillation was not considered a comorbidity. Diabetes mellitus was considered newly diagnosed if the diagnosis was set within a year prior to the cancer diagnosis. Body mass index (BMI) was calculated and grouped according to WHO [24], underweight; < 18.4, normal weight; 18.5–24.9, overweight, 25.0–29.9, Obese; > 30. Performance status score was rated by the patients’ physician according to the Eastern Cooperative Oncology Group (ECOG) scale [25]. Histopathological re-evaluation of all cases was performed by a senior pathologist (KJ). Resected tumors were classified according to WHO classification of tumors 5^th^ edition [26].

### The EORTC QLQ-C30 questionnaire

The questionnaire comprises of 30 questions and is divided into functional and symptom scales. The function scales encompass questions about physical, emotional, role, cognitive and social functioning as well as global health status (5,4,2,2,2,2 items, respectively) A high score on these scales indicate a high functional level. For symptoms there are three scales measuring nausea and vomiting, fatigue and pain, all comprising of two questions. The remaining six single questions assess various physical symptoms as well as financial impact. A high score on these scales/single questions indicates a high degree of symptoms. Before statistical analysis, the raw EORTC QLQ-C30 scores were linearly transformed to a 0–100 scale [27].

### Statistical analysis

Descriptive data is presented as numbers (n), percentages (%), mean, median and range interquartile range (IQL) as appropriate. Differences in patients included or not included in the CHAMP study, differences in treatment intention and between the sexes were evaluated by nonparametric tests, Chi-square test for categorical values and Mann–Whitney *U* for continuous variables. Univariable and multivariable logistic regression analyses were applied to calculate odds ratios for treatment allocation, sex, age and tumor location were chosen in multivariable analysis based on previous literature and performance status based on clear in univariable regression. Kaplan–Meier analysis and the log-rank test were applied to estimate survival differences. Health related quality of life (HRQoL) was assessed by scoring of EORTC-QLQ-C30 version 3 according to the EORTC QLQ-C30 Scoring Manual [23]. A *p*-value of < 0.05 was considered statistically significant. Statistical analysis was conducted using SPSS® version 27.0.1.0 (SPSS Inc®, Chicago, IL, USA).

## Results

### Demographics and clinicopathological parameters by sex and treatment intention

**Fig. 1:**
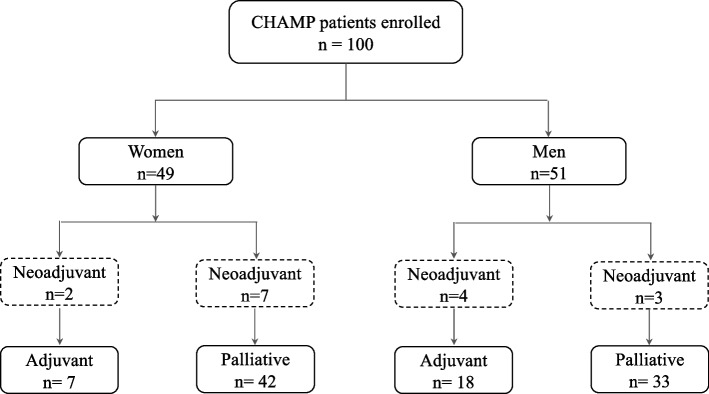
Overview of the first 100 patients enrolled in the CHAMP study. Flowchart of patients and the allocation of treatment according to sex

A flowchart of the allocation of treatment in women and men, respectively, is shown in Fig. 1. Six patients, two women and four men, received neoadjuvant chemotherapy treatment and subsequent pancreatectomy with curative intent. In comparison, ten patients, seven women and three men, were treated with neoadjuvant chemotherapy but were inoperable and went on to receive palliative treatment. In the this paper, patients who received neoadjuvant therapy (NAT) but were inoperable are stated as palliative regarding treatment intention.

Median follow-up was 22.7 (range 3.6–45.4) months for curatively treated patients and 7.8 (range 1.0–44.5) months for palliatively treated patients. Further demographic characteristics by sex and treatment intention are shown in Table 1. Seven (28%) women and 18 (72%) men were treated with curative intent, including both surgery after NAT and upfront surgery. Forty-two (56%) women and 33 (44%) men were treated with palliative intent. Two female patients were offered surgery with curative intent but declined, and were subsequently included in the patient group treated with palliative intent. Age at diagnosis did not differ significantly between the sexes in the different treatment categories. In palliative, but not adjuvant, patients, women had a significantly lower BMI than men (*p* = 0.021), and men had significantly more cardiac comorbidities (*p* = 0.031). In patients treated with adjuvant chemotherapy, significantly more women had a history of other malignancies compared to men (*p* = *0.025*). In patients treated with adjuvant intent, none of the women had a performance status of more than 1 (0/7), whereas 5/18 (28%) male patients had a performance status of 2 at start of chemotherapy, this difference did however not reach statistical significance. The five patients with ECOG 2 had undergone total pancreatectomy or Whipple procedure with various complications, two had pancreatic anastomosis leakage, two had postoperative infections, one had a hernia that required reoperation and one a splenic infarction and colitis. No differences were seen between the sexes in either treatment strata regarding age, diabetes, smoking, marital status, neoadjuvant treatment, treatment backbone or location (Table 1).

**Table 1:** Demographic characteristics by sex and treatment intention

	**Adjuvant**			**Palliative**		
	Women	Men	*P-value*^a^	Women	Men	*P-value*^a^
N (%)	7 (28)	18 (72)		42 (56)	33 (44)	
**Age** (years)						
Mean, ± SD	66.5, 9.0	68.4, 5.6	*0.745*	68.1, 11.0	68.6, 7.3	*0.741*
(Range)	(51.7- 76.2)	(55.2–77.0)		(38.4—83.3)	(49.2- 79.5)	
**ECOG at baseline**						
0	5 (71)	9 (50)	*0.173*	6 (14)	8 (24)	*0.436*
1	2 (29)	4 (22)		22 (52)	17 (52)	
2	0	5 (2)		12 (29)	5 (15)	
3	0	0		2 (5)	3 (9)	
**BMI** (kg/m2)						
Underweight	1 (14.3)	0 (0.00)	*0.154*	4 (10)	1 (3)	*0.385*
Normal weight	4 (57.1)	9 (50.0)		26 (62)	22 (67)	
Overweight	2 (28.6)	8 (44.4)		8 (19)	6 (18)	
Obese	0 (0.00)	1 (5.6)		3 (7)	4 (12)	
Mean, ± SD	23.5, 3.8	24.9, 3.2	*0.326*	22.5, 5.2	25.1, 4.5	***0.021***
(range)	(18.4–29.8)	(19.8–32.7)		(16.7–43.0)	(17.0–39.4)	
*Missing*				*1 (2)*		
**Civil Status**						
Single	0 (0)	1 (6)	*0.443*	5 (12)	3 (9)	0.697
Married	5 (71)	14 (78)		27 (64)	24 (73)	
Divorced/Widowed	2 (29)	3 (16)		10 (24)	6 (18)	
**Diabetes mellitus**						
No	4 (57.1)	13 (72)	*0.477*	36 (86)	23 (70)	*0.240*
Yes	3 (42.9)	5 (28)		4 (9)	9 (27)	
Newly diagnosed	0	0		2 (5)	1 (3)	
**Cardiac comorbidity**						
No	6 (86)	17 (94)	*0.479*	40 (95)	26 (79)	***0.031***
Yes	1 (14)	1 (6)		2 (5)	7 (21)	
**Smoking**						
No	5 (71)	7 (39)		21 (50.0)	17 (52)	
Yes, Current	2 (29)	2 (11)	***0.046***	10 (24)	5 (15)	*0.808*
Yes, Former	0	9 (50)		10 (24)	10 (30)	
*Missing*				*1 (2)*	*1 (3.)*	
**Other Cancer**						
No	4 (57)	17 (94)	***0.025***	30 (71)	28 (85)	*0.171*
Yes	3 (43)	1 (6)		12 (29)	5 (15)	
**Neoadjuvant intent**						
No	5 (71)	14 (78)	*0.744*	35 (83)	30 (91)	*0.497*
Yes	2 (29)	4 (22)		7 (17)	3 (9)	
**Chemotherapy backbone**						
Gemcitabine	2 (28.5)	8 (44)	*0.375*	8 (19)	4 (15)	*0.732*
Nabpaclitaxel	0	1 (6)		17 (40.5)	14 (42.5)	
Oxaliplatin	2 (28.5)	4 (22)		17 (40.5)	14 (42.5)	
5-FU	3 (43)	5 (28)		0	0	
**Location**						
Pancreatic head	4 (57)	13 (72)	*0.477*	23 (55)	15 (45.5)	*0.427*
Other	3 (43)	5 (28)		19 (45)	18 (54.5)	
**Tumour stage**						
Locally Advanced	-	-		18 (43)	10 (30)	*0.268*
Metastasised	-	-		24 (57)	23 (70)	

*Abbreviations*: *ECOG* Eastern Cooperative Oncology Group, *BMI* Body Mass Index, *5-FU* 5-fluoruracil

^a^Non-parametric test for continuous variables and X^2^ test for categorical variables

As further shown in Table 2, female sex was significantly associated with a decreased likelihood of being selected for curative resection in both univariable (*p* = 0.018) and multivariable analysis (*p* = 0.017) in the whole cohort. Of note, this was also the case when palliative patients with metastatic disease were excluded from the analyses in both univariable (*p* = 0.010) and multivariable analysis (*p* = 0.037), as shown in Additional file 1.

**Table 2:** Logistic regression of factors determining decision of treatment with curative intent

		**Univariable analysis**			**Multivariable Analysis**		
	Events/total	OR	95% CI	*p-value*	OR	95% CI	*p-value*
Sex (female)	49/100	0.306	0.11–0.82	*0.018*	0.294	0.11–0.81	*0.017*
Age	100/100	1.005	0.96–1.06	*0.834*	1.013	0.95–1.08	*0.671*
Location (pancreatic head)	55/100	2.069	0.80–5.37	*0.135*	1.387	0.42–4.58	*0.591*
ECOG (0–1)	73/100	1.660	0.55–4.98	*0.306*	2.192	0.79–6.06	*0.131*

*Abbreviations*: *ECOG* Eastern Cooperative Oncology Group

For the 25 patients who had undergone surgery with curative intent, no significant differences between the sexes were seen regarding tumor size, location, surgical procedure, tumor stage, number of resected lymph nodes, R status, tumor grade, vascular invasion, lymphatic invasion and perineural invasion (Table 3). Kaplan–Meier analysis showed no significant differences in OS between the sexes, neither in adjuvant nor in palliative treated patients (Additional file 2).

**Table 3:** Distribution of clinicopathological parameters in resected tumors by sex

	**Women**	**Men**
N(%)	7 (18%)	18 (72%)
**Tumour size mm**		
Median (range)	25.5 (10.0–33.0)	28.5 (14.0–45.0)
*Missing*	*1*	*2*
**Tumour location**		
Caput	4 (57.1)	16 (77.7)
Corpus	1 (14.3)	1 (5.6)
Cauda	2 (28.6)	2 (11.1)
Other^a^	0	2 (11.1)
**T stage**		
T1-T2	5 (71.4)	15 (83.3)
T3-T4	2 (28.6)	3 (16.7)
**Resected lymph nodes**		
< 15	3 (42.9)	4 (22.2)
> 15	4 (57.1)	14 (77.8)
**Metastatic lymph nodes **		
0	1 (14.3)	7 (38.9)
1–3	2 (28.6)	6 (33.3)
> 3	3 (57.1)	5 (27.8)
**R status**		
0	4 (57.1)	7 (38.9)
1	3 (42.9)	11 (61.1)
**Tumour Grade**		
Well-moderate	1 (14.3)	3 (16.7)
Poor	4 (57.1)	8 (44.4)
*Missing*	*2 (28.6)*	*7 (38.9)*
**Vascular invasion**		
Absent	3 (42.9)	13 (72.2)
Present	4 (57.1)	5 (27.8)
**Lymphatic invasion**		
Absent	3 (42.9)	9 (50.0)
Present	4(57.1)	9 (50.0)
**Perineural invasion**		
Absent	2 (28.6)	6 (33.3)
Present	5 (71.4)	12 (66.7)
**Surgical procedure**		
Whipple	4 (57.1)	15 (83.3)
Distal pancreatectomy	3 (42.9)	3 (16.7)

^a^1 Distal cholangiocarcinoma, 1 ampullary adenocarcinoma. Non-parametric test for continuous variables and X^2^ test for categorical variables

### Quality of Life

Seventy-five patients, 42 men and 33 women, had completed EORTC QLQ-C30 questionnaires from baseline. The demographic characteristics of patients with and without completed questionnaires stratified by treatment intention are shown in Additional file 3, and no significant differences were seen. The distribution of measurements of HRQoL in women and men independently of treatment intention is shown in Table 4. Female patients experienced significantly poorer emotional functioning (*p* = 0.011) and cognitive functioning (*p* = 0.013) than male patients at the start of treatment. Female patients were also subject to more severe symptoms such as fatigue (*p* = 0.020), nausea/vomiting (*p* =  < 0.001), insomnia (*p* = 0.017) and loss of appetite (*p* = 0.004). Of note, no differences were seen between the sexes regarding physical, role and social functioning, pain or global health score.

**Table 4:** Health related quality of life by sex

	**Women**	**Men**	***P-value***^a^
N	49	51	
**Global Health Score**			
Median (IQR)	42 (0–92)	58 (0–100)	*0.271*
*Missing*	*16*	*11*	
**Physical functioning**			
Median (IQR)	73 (53–80)	80 (60–88)	*0.075*
*Missing*	*16*	*9*	
**Role functioning**			
Median (IQR)	50 (33–67)	67 (33–88)	*0.076*
*Missing*	*16*	*9*	
**Emotional functioning**			
Median (IQR)	58 (50–75)	71 (67–92)	***0.011***
*Missing*	*16*	*11*	
**Cognitive functioning**			
Median (IQR)	83 (58–100)	100 (71–100)	***0.013***
*Missing*	*16*	*11*	
**Social functioning**			
Median(IQR)	50.0 (33–75)	67 (50–83)	*0.243*
*Missing*	*16*	*11*	
**Fatigue**			
Median (IQR)	56 (33–78)	33 (33–56)	***0.020***
*Missing*	*16*	*9*	
**Nausea and vomiting**			
Median (IQR)	17 (0–33)	0 (0–17)	** < *0.001***
*Missing*	*16*	*9*	
**Pain**			
Median (IQR)	33 (17–83)	33 (13–50)	*0.164*
*Missing*	*16*	*9*	
**Dyspnea**			
Median (IQR)	0 (0–33)	33 (0–33)	*0.872*
*Missing*	*16*	*9*	
**Insomnia**			
Median (IQR)	33 (0–68)	17 (0–33)	***0.017***
*Missing*	*16*	*9*	
**Loss of appetite**			
Median (IQR)	67 (33–100)	33 (0–42)	***0.004***
*Missing*	*16*	*9*	
**Constipation**			
Median (IQR)	33 (0–67)	33 (0–33)	*0.125*
*Missing*	*16*	*9*	
**Diarrhea**			
Median (IQR)	0 (0–50)	0 (0.0–33)	*0.750*
*Missing*	*16*	*11*	
**Financial difficulties**			
Median (IQR)	0 (0–0)	0 (0–0)	*0.886*
*Missing*	*18*	*9*	

*Abbreviation*: *IQR* interquartile range

^a^Non-parametric test for continuous variables

When stratifying by treatment intention alone, adjuvant patients had a significantly higher global health score (*p* = 0.024) as well as better physical, role, emotional and social functioning (*p* =  < 0.001, < 0.001, 0.025, < 0.001, respectively). Adjuvant patients also had lower levels of fatigue (*p* < 0.001), less nausea (*p* = 0.036), less pain (*p* = 0.001), and a better appetite (*p* < 0.001) than palliative patients (Additional file 4).

When stratifying patients by sex and treatment intention, no significant difference in quality of life was noted in the small number of patients within each group. The biggest discrepancies between the sexes, although non-significant due to the small sample size, were seen in the adjuvant group of patients with a more than a 20-point median difference in certain scores. Female patients seemingly had more pain (score 33 vs. 0), a larger loss of appetite (score 67 vs.0) and poorer role functioning (score 50 vs. 100) as well as emotional functioning (score 50–83) than male patients (Additional file 5).

Correlations between performance status and HRQoL at baseline in female and male patients, respectively, are shown in Table 5. Of note, in female patients, there was no significant decrease in any HRQoL parameter with decreased performance status. Contrastingly, for male patients, a poorer performance status correlated significantly with a poorer experienced physical function (*p* = 0.002), emotional functioning (*p* = 0.038), social functioning (*p* = 0.022) as well as a higher level of fatigue (*p* < 0.001). As seen in Additional file 6 these correlations remained significant when dividing performance status into two groups (ECOG 0–1 vs 2–3).

**Table 5:** Health related quality of life by performance status and sex

	**Female**				***P-value***	**Male**				***P-value***^a^
**ECOG**	0	1	2	3		0	1	2	3	
**Global Health Score**										
Median score	63	42	50	58	*0.572*	67	54	50	63	*0.136*
N (%)	6 (18.8)	18 (56.3)	6 (18.8)	2 (6.3)		13 (32.5)	16 (40.0)	9 (22.5)	2 (5.0)	
**Physical functioning**										
Median score	80	63	53	73	*0.062*	90	73	67	50	***0.002***
N (%)	6 (18.8)	18 (56.3)	6 (18.8)	2 (6.3)		14 (33.3)	17 (40.5)	9 (21.4)	2 (4.8)	
**Role functioning**										
Median score	67	33	50	58	*0.336*	92	50	67	58	*0.058*
N (%)	6 (18.8)	18 (56.3)	6 (18.8)	2 (6.3)		14 (33.3)	17 (40.5)	9 (21.4)	2 (4.8)	
**Emotional functioning**										
Median score	63	63	58	50	*0.594*	83	67	67	46	***0.038***
N (%)	6 (18.8)	18 (56.3)	6 (18.8)	2 (6.3)		13 (32.5)	16 (40.0)	9 (22.5)	2 (5.0)	
**Cognitive functioning**										
Median score	92	83	67	83	*0.583*	100	100	83	67	*0.157*
N (%)	6 (18.8)	18 (56.3)	6 (18.8)	2 (6.3)		13 (32.5)	16 (40.0)	9 (22.5)	2 (5.0)	
**Social functioning**										
Median score	50	50	50	83	*0.525*	67	67	67	33	***0.022***
N (%)	6 (18.8)	18 (56.3)	6 (18.8)	2 (6.3)		13 (32.5)	16 (40.0)	9 (22.5)	2 (5.0)	
**Fatigue**										
Median score	44	61	78	39	*0.077*	33	44	56	56	** < *0.001***
N (%)	6 (18.8)	18 (56.3)	6 (18.8)	2 (6.3)		14 (33.3)	17 (40.5)	9 (21.4)	2 (4.8)	
**Nausea**										
Median score	8	17	17	8	*0.503*	0	0	0	17	*0.474*
N (%)	6 (18.8)	18 (56.3)	6 (18.8)	2 (6.3)		14 (33.3)	17 (40.5)	9 (21.4)	2 (4.8)	
**Pain**										
Median score	33	42	33	33	*0.784*	25	50	33	50	*0.265*
N (%)	6 (18.8)	18 (56.3)	6 (18.8)	2 (6.3)		14 (33.3)	17 (40.5)	9 (21.4)	2 (4.8)	
**Dyspnea**										
Median score	0	17	33	33	*0.217*	0	33	33	33	*0.078*
N (%)	6 (18.8)	18 (56.3)	6 (18.8)	2 (6.3)		14 (33.3)	17 (40.5)	9 (21.4)	2 (4.8)	
**Insomnia**										
Median score	33	33	0	66.7	*0.626*	33	0	0	33	*0.418*
N (%)	6 (18.8)	18 (56.3)	6 (18.8)	2 (6.3)		14 (33.3)	17 (40.5)	9 (21.4)	2 (4.8)	
**Loss of appetite**										
Median score	50	67	67	50	*0.266*	0	33	33	33	*0.111*
N (%)	6 (18.8)	18 (56.3)	6 (18.8)	2 (6.3)		14 (33.3)	17 (40.5)	9 (21.4)	2 (4.8)	
**Constipation**										
Median score	0	33	33	50	*0.823*	17	33	0	17	*0.594*
N (%)	6 (18.8)	18 (56.3)	6 (18.8)	2 (6.3)		14 (33.3)	17 (40.5)	9 (21.4)	2 (4.8)	
**Diarrhea**										
Median score	17	17	0	0	*0.312*	0	17	0	33	*0.371*
N (%)	6 (18.8)	18 (56.3)	6 (18.8)	2 (6.3)		13 (32.5)	16 (40.0)	9 (22.5)	2 (5.0)	
**Financial difficulties**										
Median score	0	0	0	17	*0.493*	0	0	0	0	*0.493*
N (%)	6 (18.8)	17 (53.1)	7 (21.9)	2 (6.3)		13 (32.5)	16 (40.0)	9 (22.5)	2 (5.0)	

For functional scores, a high score indicates a high functional level, for symptom scores a high value indicates an increased severity of symptoms

*Abbreviation*: *ECOG* Eastern Cooperative Oncology Group

^a^Non-parametric test for continuous variables

## Discussion

The influence of sex and gender on the choice of treatment and the HRQoL of patients with pancreatic and other periampullary cancers has hitherto been little studied, but it is evident that increased awareness of these issues is vital in order to prevent gender-based bias and to achieve optimized personalized treatment. This study of the first 100 patients with periampullary adenocarcinoma enrolled in the CHAMP study shows a significant difference between men and women regarding treatment with curative intent, with less women having undergone pancreatectomy. This is in line with the findings of a previous nationwide Swedish study of patients with periampullary adenocarcinoma [8], although in that study, the significant difference between sexes was lost after adjusting for age and tumor location, with female patients being of older age and having more tumors located in the pancreatic head. In contrast, in the current study, female patients were slightly younger than male patients (66.5 vs. 68.4 years of age) and fewer women had tumors located in the pancreatic head. Moreover, no patients with a periampullary tumor originating in the duodenum has been included in the CHAMP study, since these patients receive a different chemotherapy regimen.

It has also been shown that male patients have a higher morbidity rate than female patients after pancreatectomy and that female patients with early-stage pancreatic cancer have prolonged survival after pancreatectomy [8, 28]. Our results showed no significant difference in OS between men and women after curative surgery, but a larger number of cases and a longer follow-up is needed. Also, male patients who underwent surgery tended to have a poorer performance status compared to female patients, although these findings did not reach statistical significance.

Patients with pancreatic cancer generally have an impaired HRQoL, although one study found no significant difference in the scales cognitive function or pain for female patients with pancreatic cancer compared to the general population [29]. It is also known that both short and long-term HRQoL decreases after pancreatectomy with no clear difference between the sexes [30, 31]. In this study, females generally had a decreased HRQoL compared to males, both in functional scales and symptoms before the start of treatment. Female patients also tended to experience more pain and had poorer social functioning than males before the start of adjuvant treatment. These findings are in line with the literature regarding cancer patients but is also true for women in the general population [32, 33]. Therefore, when interpreting cancer patients’ HRQoL it is important to always consider the reference population. In one study on the HRQoL of over 5000 long-term cancer survivors, adjustment to the reference population highlighted a significant and unexpected impact on male patients [34]. The finding in the present study that operated patients had higher HRQoL than palliative patients is not surprising but indicates that patients seemingly experienced a high level of recovery 6–8 weeks after extensive surgery, i.e. at the start of adjuvant chemotherapy treatment.

Since our results showed no statistical differences between the sexes regarding age, clinicopathological factors, or comorbidities, it is feasible to assume that gender plays an important role in the discrepancy of surgery given with curative intent. Of the 51 female patients included in this study, two were eligible for pancreatectomy but declined surgery and, hence, a chance of cure. It has been shown in several studies that patients with early-stage pancreatic cancer who decline surgery are generally of female sex, of older age, and/or suffer from more comorbidities [35–37]. Symptom perception theory hypothesizes that men and women perceive and report symptoms differently due to differences in early socialization, social position, and traditional gender roles [38]. Few studies have investigated the potential associations between patient-reported HRQoL and physicians’ assessment of performance status, and even fewer have examined this association in relation to sex or gender. In one study including 115 cancer patients with a variety of diagnoses and only six gastrointestinal cancers, female sex was found to be associated with decreased HRQoL and performance status [39]. These findings are in contrast to our results, but comparisons are difficult to make given the quite diverging study populations. Men in the general population, as well as in our study, experience a better general HRQoL than women. The finding that men with poor performance status suffer from greater fatigue and a decrease in physical, emotional, and social functioning while women’s HRQoL remains unchanged with decreasing performance status is however novel and must be attributed to the complexity of gender differences. Our understanding of the impact that gender dimensions might have on HRQoL is limited, with no scales in clinical use to date. Gender is multidimensional and while many studies primarily focus on gender identity, other gender dimensions such as gender roles, behaviors and relations, should also be investigated, as they might well be more important for HRQoL. In a recent study of patients with Parkinson’s disease no associations were found between self-reported gender identity and overall HRQoL whereas an androgynous gender role orientation and higher engagement in household tasks were associated with increased HRQoL [40]. In the previously mentioned study of > 5000 long-term cancer survivors it was concluded that men had a significant loss of social and role functioning, perhaps an indication of the loss of gender role [34].

The strengths of the present study are that it is an ongoing, prospective trial with real-world data from patients with pancreatic and other periampullary cancer, making biased patient selection minimal. The limitations include a small patient number, in particular in the adjuvant group. Only 75 patients had completed EORTC-C30 questionnaires, however no significant demographic differences were seen between patients with or without completed forms. Other limitations are that we did not adjust HRQoL for the reference population and did not use tools to analyze gender dimensions or compare patients enrolled in the CHAMP study to all patients with periampullary cancer receiving chemotherapy during this time span. Since all patients included in the CHAMP study received chemotherapy, no information was available regarding surgically treated patients who may have been unfit for adjuvant chemotherapy due to post-operative complications.

## Conclusions

The results from this study further underline the importance of gender with regard to the interaction between healthcare providers and patients, and how this interaction may affect the outcome for patients with periampullary adenocarcinoma. In this context, particular attention should be given to the selection of women for curative surgery, where a more encouraging approach might well lead to improved survival rates. The gender disparities regarding the relationship between self-perceived HRQoL and the physicians’ subjective assessment of the patients’ performance status are noteworthy, and a heightened awareness of how these factors may influence treatment decisions should be an important part of personalized medicine. In future studies, the multidimensionality of gender needs further consideration in order to gain insights into how it impacts the biological outcome for all patients.

## Supplementary Information

**Additional file 1.** Factors determining decision of treatment with curative intent in patients with non-metastatic disease. Logistic regression of operated patients and patients with locally advanced disease.

**Additional file 2.** Sex-specific survival in adjuvant and palliative treated patients. Kaplan-Meier analyses of overall survival in strata according to sex.

**Additional file 3.** Demographic comparisons of patients with and without completed EORTC QLQ-C30 questionnaires. Non-parametric test was applied for continuous variables and X^2^ test for categorical variables. No significant differences were seen between the groups.

**Additional file 4.** Health related quality of life by given treatment. Non-parametric test applied for continuous variables. For functional scores, a high score indicates a high functional level, for symptom scores a high value indicates an increased severity of symptoms.

**Additional file 5.** Health related quality of life by sex and treatment intention. Non-parametric test applied for continuous variables.

**Additional file 6.** Health related quality of life by grouped performance status and sex. Non-parametric test applied test for continuous variables. For functional scores, a high score indicates a high functional level, for symptom scores a high value indicates an increased severity of symptoms.

## Data Availability

All the data generated in this study are included in the article. Access to all data generated or analyzed during this current study will be evaluated according to Swedish legislation and be made available from the corresponding author on reasonable request.

## Abbreviations

CHAMP: Chemotherapy, Host response And Molecular dynamics in Periampullary cancer
ECOG: Eastern Cooperative Oncology Group
EORTC-QLQ-C30: The European Organisation for Research and Treatment of Cancer Quality of life Questionnaire
FOLFIRINOX: Folinic acid, 5-fluoruracil, irinotecan and oxaliplatin
HRQoL: Health related quality of life
IQR: Interquartile range
OS: Overall survival
