# Supplementary material for: Sex and gender differences in treatment intention, quality of life and performance status in the first 100 patients with periampullary cancer enrolled in the CHAMP study
Source: BMC Cancer. 2023 Apr 11;23:334. doi: 10.1186/s12885-023-10720-w (PMC10088105; doi:10.1186/s12885-023-10720-w)
Supplement: Supplementary file 1 — Additional file 1. Factors determining decision of treatment with curative intent in patients with non-metastatic disease. Logistic regression of operated patients and patients with locally advanced disease. [file 12885_2023_10720_MOESM1_ESM.docx]

**Additional File 1. Logistic regression of factors determining decision of treatment with curative intent in patients with non-metastatic disease.**

|  | | **Univariable analysis** | | | **Multivariable Analysis** | | |
| --- | --- | --- | --- | --- | --- | --- | --- |
|  | Events/total | OR | 95 % CI | *p-value* | OR | 95 % CI | *p-value* |
| Sex (female) | 18/53 | 0.216 | 0.067-0.693 | *0.010* | 0.274 | 0.08-0.93 | *0.037* |
| Age | 53/53 | 1.033 | 0.965-1.107 | *0.348* | 1.037 | 1.04-0.96 | *0.391* |
| Location (pancreatic head) | 32/53 | 3.654 | 1.122-11.899 | *0.031* | 3.265 | 3.27-0.90 | *0.071* |
| ECOG (0-1) | 41/53 | 1.333 | 0.363-5.896 | *0.665* | 0.903 | 0.90-0.17 | *0.903* |

Abbreviations: ECOG; Eastern Cooperative Oncology Group.
