# Supplementary figures and images for: Sex and gender differences in treatment intention, quality of life and performance status in the first 100 patients with periampullary cancer enrolled in the CHAMP study
Source: BMC Cancer. 2023 Apr 11;23:334. doi: 10.1186/s12885-023-10720-w (PMC10088105; doi:10.1186/s12885-023-10720-w)

Adjuvant

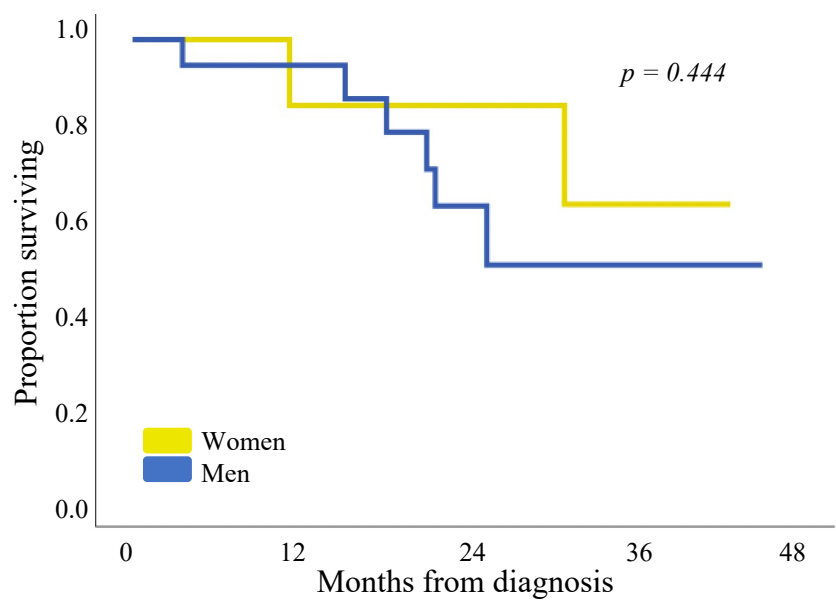

Number at risk

|       |    |    |    |    |
|-------|----|----|----|----|
| Women | 7  | 6  | 0  | 5  |
| Men   | 18 | 17 | 13 | 12 |

Palliative

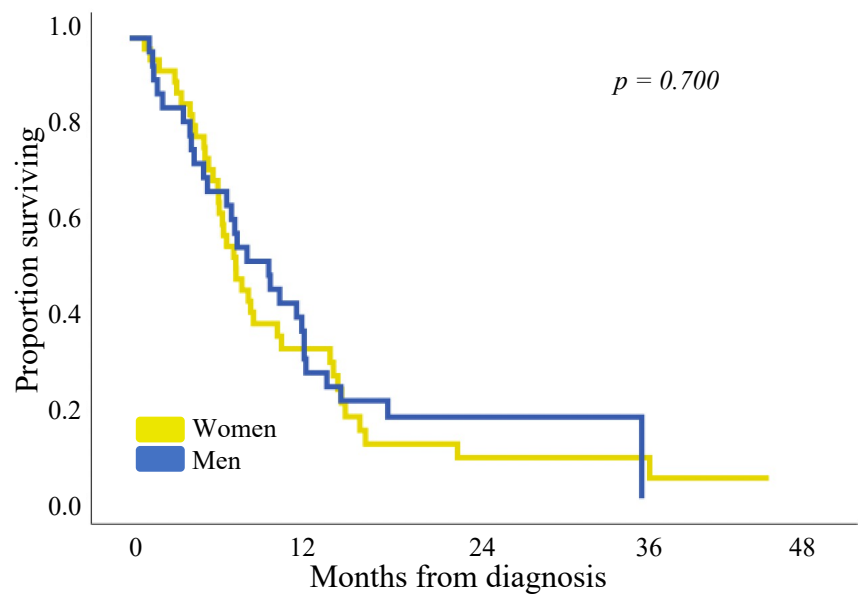

Number at risk

|       |    |    |   |   |
|-------|----|----|---|---|
| Women | 42 | 14 | 6 | 6 |
| Men   | 33 | 12 | 6 | 5 |

Supplement: Supplementary file 2 — Additional file 2. Sex-specific survival in adjuvant and palliative treated patients. Kaplan-Meier analyses of overall survival in strata according to sex. [file 12885_2023_10720_MOESM2_ESM.pdf]
