# Supplementary material for: Sex and gender differences in treatment intention, quality of life and performance status in the first 100 patients with periampullary cancer enrolled in the CHAMP study
Source: BMC Cancer. 2023 Apr 11;23:334. doi: 10.1186/s12885-023-10720-w (PMC10088105; doi:10.1186/s12885-023-10720-w)
Supplement: Supplementary file 3 — Additional file 3. Demographic comparisons of patients with and without completed EORTC QLQ-C30 questionnaires. Non-parametric test was applied for continuous variables and X2 test for categorical variables. No significant differences were seen between the groups. [file 12885_2023_10720_MOESM3_ESM.docx]

**Additional file 3. Demographic comparison of patients with and without completed EORTC QLQ-C30 questionnaires.**

|  | Adjuvant | | | | Palliative | | | |
| --- | --- | --- | --- | --- | --- | --- | --- | --- |
|  | Women with QoL data | Women without QoL data | Men with QoL data | Men without QoL data | Women with QoL data | Women without QoL data | Men with QoL data | Men without QoL data |
| N (%) | 3 (43) | 4 (57) | 15 (83) | 3 (17) | 30 (71) | 12 (29) | 27 (82) | 6 (18) |
| **Age** (years) |  |  |  |  |  |  |  |  |
| Mean, SD± | 67.6, 3.5 | 65.7, 12.3 | 67.7, 6.2 | 72.3, 4.6 | 68.0, 10.0 | 68.4, 13.0 | 69.4, 7.0 | 65.2, 8.4 |
| (Range) | 63.6-70.3 | 51.7-76.2 | 55.2-77.0 | 69.2-75.1 | 39.2-83.3 | 38.4-82.7 | 55.9-79.5 | 49.2-74.4 |
| **ECOG** |  |  |  |  |  |  |  |  |
| 0 | 2 (67) | 3 (75) | 8 (53) | 1 (33.3) | 4 (13) | 2 (16) | 6 (22) | 2 (33) |
| 1 | 1(33) | 1 (25) | 3 (20) | 1 (33.3) | 17 (57) | 5 (42) | 14 (52) | 3 (50) |
| 2 | - | - | 4 (27) | 1 (33.3) | 7 (23) | 5 (42) | 5 (17) |  |
| 3 | - | - | - | - | 2 (7) | - | 2 (7) | 1 (16) |
| **BMI** (kg/m2) |  |  |  |  |  |  |  |  |
| Underweight | 1 (33) | - | - | *-* | 3 (10) | 1 (8) | 1 (4) | - |
| Normal weight | 1 (33) | 3 (75) | 7 (47) | 2 (67) | 18 (60) | 8 (67) | 18 (67) | 4 (67) |
| Overweight | 1 (33) | 1 (25) | 7 (47) | 1 (33) | 6 (20) | 2 (167) | 5 (19) | 1 (17) |
| Obese | - | - | 1 (7) | *-* | 2 (7) | 1 (8) | 3 (11) | 1 (17) |
| Mean, SD±  (range) | 21.2, 3.9  18.4-25.6 | 25.2, 3.1  23.1-29.8 | 24.9, 3.5  19.8-32.7 | 24.8, 2.7  24.5-25.2 | 23.7, 5.8  16.7-43.0 | 23.0, 3.8  16.8-31.9 | 24.8, 3.6  17.0-36.5 | 26.6, 6.1  22.9-39.4 |
| *Missing* |  |  |  |  | *1 (3.3)* |  |  |  |
| **Civil Status** |  |  |  |  |  |  |  |  |
| Single | - | - | - | 1 (33) | 4 (13) | 1 (8) | 1 (4) | 2 (33) |
| Married | 3 (100) | 2 (50) | 13 (87) | 1 (33) | 21 (70) | 6 (50) | 21 (78) | 3 (50) |
| Divorced/Widowed | - | 2 (50) | 2 (13) | 1 (33) | 5 (17) | 5 (42) | 5 (19) | 1 (17) |
| **Diabetes mellitus** |  |  |  |  |  |  |  |  |
| No | 2 (67) | 2 (50) | 11 (73) | 2 (67) | 24 (80) | 12 (100) | 19 (70) | 4 (67) |
| Yes | 1 (33) | 2 (50) | 4 (27) | 1 (33) | 4 (13) | - | 7 (26) | 2 (33) |
| Newly diagnosed | - | - | - | - | 2 (7) | - | 1 (4) | - |
| **Cardiac comorbidity** |  |  |  |  |  |  |  |  |
| No | 3 (100) | 3 (75) | 14 (93) | 3 (100) | 28 (93) | 12 (100) | 21 (78) | 5 (83) |
| Yes | - | 1 (25) | 1 (7) | - | 2 (7) | - | 6 (22) | 1 (17) |
| **Smoking** |  |  |  |  |  |  |  |  |
| No | 3 (100) | 2 (50) | 6 (40) | 1 (33) | 14 (47) | 7 (58) | 13 (58) | 4 (67) |
| Yes, Current | - | 2 (50) | 2 (13) | - | 8 (27) | 2 (17) | 5 (19) |  |
| Yes, Former | - | - | 7 (47) | 2 (67) | 7 (23) | 3 (25) | 8 (30) | 2 (33) |
| *Missing* |  |  |  |  | *1 (3)* |  | *1 (4)* |  |
| **Other Cancer** |  |  |  |  |  |  |  |  |
| No | 2 (67) | 2 (50) | 14 (93) | 3 (100) | 21 (70) | 9 (75) | 22 (82) | 6 (100) |
| Yes | 1 (33) | 2 (50) | 1 (7) | - | 9 (30) | 3 (25) | 5 (19) | - |
| **Neoadjuvant intent** |  |  |  |  |  |  |  |  |
| No | 2 (67) | 3 (75) | 11 (73) | 3 (100) | 26 (87) | 9 (75) | 24 (89) | 6 (100) |
| Yes | 1 (33) | 1 (25) | 4 (27) | - | 4 (13) | 3 (25) | 3 (11) | - |
| **Chemotherapy backbone** |  |  |  |  |  |  |  |  |
| Gemcitabine | - | 2 (50) | 5 (33) | 3 (100) | 5 (17) | 3 (25) | 4 (15) | 1 (17) |
| Nabpaclitaxel | - | - | 1 (7) | - | 13 (43) | 4 (33) | 12 (44) | 2 (33) |
| Oxaliplatin | - | 2 (50) | 4 (27) | - | 12 (40) | 5 (42) | 11 (41) | 3 (50) |
| 5-FU | 3 (100) | - | 5 (33) | - | - | - | - | - |
| **Location** |  |  |  |  |  |  |  |  |
| Pancreatic head | 2 (67) | 2 (50) | 10 (67) | 3 (100) | 16 (53) | 7 (58) | 13 (48) | 2 (33) |
| Other | 1 (33) | 2 (50) | 5 (33) |  | 14 (47) | 5 (42) | 14 (52) | 4 (67) |

Non-parametric test for continuous variables and X^2^ test for categorical variables. Abbreviations: ECOG; Eastern Cooperative Oncology Group. BMI; Body Mass Index. 5-FU; 5-fluoruracil.
