# Supplementary material for: Sex and gender differences in treatment intention, quality of life and performance status in the first 100 patients with periampullary cancer enrolled in the CHAMP study
Source: BMC Cancer. 2023 Apr 11;23:334. doi: 10.1186/s12885-023-10720-w (PMC10088105; doi:10.1186/s12885-023-10720-w)
Supplement: Supplementary file 4 — Additional file 4. Health related quality of life by given treatment. Non-parametric test applied for continuous variables. For functional scores, a high score indicates a high functional level, for symptom scores a high value indicates an increased severity of symptoms. [file 12885_2023_10720_MOESM4_ESM.docx]

**Additional file 4. Health related quality of life by given treatment.**

|  | **Adjuvant** | **Palliative** | ***P-value**** |
| --- | --- | --- | --- |
| N | 25 | 75 |  |
| **Global Health Score**  Median (IQR)  *Missing* | 63 (33-100)  *7* | 50 (0-92)  *20* | ***0.024*** |
| **Physical functioning**  Median (IQR)  *Missing* | 87 (73-100)  *7* | 73 (53-80)  *18* | ***<0.001*** |
| **Role functioning**  Median (IQR)  *Missing* | 92 (63-100)  *7* | 50 (33-68)  *18* | ***<0.001*** |
| **Emotional functioning**  Median (IQR)  *Missing* | 83 (65-94)  7 | 67 (50-83)  *20* | ***0.025*** |
| **Cognitive functioning**  Median (IQR)  *Missing* | 100 (79-100)  7 | 83 (67-100)  *20* | *0.195* |
| **Social functioning**  Median (IQR)  *Missing* | 67 (63-100)  *7* | 50 (33-67)  *20* | ***0.009*** |
| **Fatigue**  Median (IQR)  *Missing* | 33 (17-47)  *7* | 56 (33-78)  *18* | ***<0.001*** |
| **Nausea**  Median (IQR)  *Missing* | 0 (0-17)  *7* | 17 (0-25)  *18* | ***0.036*** |
| **Pain**  Median (IQR)  *Missing* | 0 (0-38)  *7* | 50 (25-67)  *18* | ***0.001*** |
| **Dyspnea**  Median (IQR)  *Missing* | 0 (0-33)  7 | 33 (0-33)  *18* | *0.175* |
| **Insomnia**  Median (IQR)  *Missing* | 0 (0-33)  *7* | 50 (0-33)  *18* | *0.213* |
| **Loss of appetite**  Median (IQR)  *Missing* | 0 (0-33)  *7* | 67 (33-100)  *18* | ***<0.001*** |
| **Constipation**  Median V  *Missing* | 0 (0-33)  *7* | 33 (0-33)  *18* | *0.246* |
| **Diarrhea**  Median (IQR)  *Missing* | 0 (0-33)  *7* | 0 (0-33)  *20* | *0.545* |
| **Financial difficulties**  Median (IQR)  *Missing* | 0 (0-0)  *7* | 0 (0-0)  *22* | *0.294* |

*Non-parametric test for continuous variables. For functional scores, a high score indicates a high functional level, for symptom scores a high value indicates an increased severity of symptoms. Abbreviation: IQR; interquartile range.
