# Supplementary material for: Sex and gender differences in treatment intention, quality of life and performance status in the first 100 patients with periampullary cancer enrolled in the CHAMP study
Source: BMC Cancer. 2023 Apr 11;23:334. doi: 10.1186/s12885-023-10720-w (PMC10088105; doi:10.1186/s12885-023-10720-w)
Supplement: Supplementary file 5 — Additional file 5. Health related quality of life by sex and treatment intention. Non-parametric test applied for continuous variables. [file 12885_2023_10720_MOESM5_ESM.docx]

**Additional file 5. Health related quality of life by sex and treatment intention.**

|  | **Adjuvant** | | **Palliative** | |
| --- | --- | --- | --- | --- |
|  | **Women** | **Men** | **Women** | **Men** |
| N (%) | 7 (28.0%) | 18 (72.0%) | 42 (56.0) | 33 (44.0) |
| **Global Health Score**  Median (IQR)  *Missing* | 58 (42-92) *  *4* | 67 (33-100)  *3* | 42 (0-92)  *12* | 58 (0-83)  *8* |
| **Physical functioning**  Median (IQR)  *Missing* | 73.3 (53-93)  *4* | 86.7 (73-87)  3 | 70 (53-80)  *12* | 63 (53-87)  *6* |
| **Role functioning**  Median (IQR)  *Missing* | 50 (33-100)  *4* | 100 (67-100)  3 | 50 (33-67)  *12* | 50 (33-83)  *6* |
| **Emotional functioning**  Median (IQR)  *Missing* | 50 (50-100)  *4* | 83 (67-92)  3 | 58 (50-75)  *12* | 67 (61-88)  *8* |
| **Cognitive functioning**  Median (IQR)  *Missing* | 83 (67-100)  *4* | 100 (83-100)  3 | 83 (50-100)  *12* | 83 (67-100)  *8* |
| **Social functioning**  Median (IQR)  *Missing* | 50 (33-100)  *4* | 67 (67-100)  3 | 50 (33-71)  *12* | 67 (33-67)  *8* |
| **Fatigue**  Median (IQR)  *Missing* | 22 (22-56)  *4* | 33 (0-44)  3 | 61 (42-78)  *12* | 44 (33-67)  *6* |
| **Nausea**  Median (IQR)  *Missing* | 0 (0-33)  *4* | 0 (0-17)  3 | 17 (13-33)  *12* | 0 (0-17)  *6* |
| **Pain**  Median (IQR)  *Missing* | 33 (0-100)  *4* | 0.0 (0-17)  3 | 33 (17-83)  *12* | 50 (33-50)  *6* |
| **Dyspnea**  Median (IQR)  *Missing* | 0 (0-33)  *4* | 0 (0-33)  3 | 17 (0-33)  *12* | 33 (0-33)  *6* |
| **Insomnia**  Median (IQR)  *Missing* | 0 (0-100)  *4* | 33 (0-33)  3 | 33 (0-67)  *12* | 0 (0-33)  *6* |
| **Loss of appetite**  Median (IQR)  *Missing* | 67 (0-100)  *4* | 0 (0-33)  3 | 67 (33-100)  *12* | 33 (33-100)  *6* |
| **Constipation**  Median (IQR)  *Missing* | 0 (0-0)  *4* | 33 (0-33)  3 | 33 (0-67)  *12* | 33 (0-33)  *6* |
| **Diarrhea**  Median (IQR)  *Missing* | 33 (0-33)  *4* | 0 (0-33)  3 | 0 (0-67)  *12* | 0 (0-33)  *8* |
| **Financial difficulties**  Median (IQR)  *Missing* | 0 (0-0)  *4* | 0 (0-33)  *3* | 0 (0-0)  *14* | 0 (0-0)  *8* |

*Range for women in the adjuvant group due to small sample size. For functional scores, a high score indicates a high functional level, for symptom scores a high value indicates an increased severity of symptoms. Abbreviation: IQR; interquartile range.
