# Supplementary material for: Sex and gender differences in treatment intention, quality of life and performance status in the first 100 patients with periampullary cancer enrolled in the CHAMP study
Source: BMC Cancer. 2023 Apr 11;23:334. doi: 10.1186/s12885-023-10720-w (PMC10088105; doi:10.1186/s12885-023-10720-w)
Supplement: Supplementary file 6 — Additional file 6. Health related quality of life by grouped performance status and sex. Non-parametric test applied test for continuous variables. For functional scores, a high score indicates a high functional level, for symptom scores a high value indicates an increased severity of symptoms. [file 12885_2023_10720_MOESM6_ESM.docx]

**Additional file 6.** **Health related quality of life by grouped performance status and sex.**

|  | **Female** | | ***P-value*** | **Male** | | ***P-valu****e^*^* |
| --- | --- | --- | --- | --- | --- | --- |
| **ECOG** | 0-1 | 2-3 |  | 0-1 | 2-3 |  |
| **Global Health Score**  Median score  N (%) | 42  24 (73) | 50  9 (27) | *0.991* | 58  29 (72) | 50  11 (28) | *0.284* |
| **Physical functioning**  Median score  N (%) | 73  24 (73) | 60  9 (27) | *0.132* | 87  31 (74) | 67  11 (26) | ***0.031*** |
| **Role functioning**  Median score  N (%) | 50  24 (73) | 50  9 (27) | *0.696* | 67  31 (74) | 67  11 (26) | *0.104* |
| **Emotional functioning**  Median score  N (%) | 63  24 (73) | 58  9 (27) | *0.229* | 83  29 (72) | 67  11 (28) | ***0.015*** |
| **Cognitive functioning**  Median score  N (%) | 83  24 (73) | 83  9 (27) | *0.428* | 100  29 (72) | 67  11 (28) | *0.061* |
| **Social functioning**  Median score  N (%) | 50  24 (73) | 50  9 (27) | *0.620* | 67  29 (72) | 50  11 (28) | ***0.010*** |
| **Fatigue**  Median score  N (%) | 56  24 (73) | 67  9 (27) | *0.306* | 33  31 (74) | 56  11 (26) | ***0.002*** |
| **Nausea**  Median score  N (%) | 17  24 (73) | 17  9 (27) | *0.488* | 0  31 (74) | 0  11 (26) | *0.743* |
| **Pain**  Median score  N (%) | 33  24 (73) | 33  9 (27) | *0.837* | 33  31 (74) | 50  11 (26) | *0.346* |
| **Dyspnea**  Median score  N (%) | 0  24 (73) | 33  9 (27) | *0.114* | 0  31 (74) | 33  11 (26) | *0.096* |
| **Insomnia**  Median score  N (%) | 33  24 (73) | 33  9 (27) | *0.833* | 33  31 (74) | 0  11 (26) | *0.423* |
| **Loss of appetite**  Median score  N (%) | 67  24 (73) | 67  9 (27) | *0.328* | 33  31 (74) | 33  11 (26) | *0.234* |
| **Constipation**  Median score  N (%) | 17  24 (73) | 33  9 (27) | *0.362* | 33  31 (74) | 0  11 (26) | *0.281* |
| **Diarrhea**  Median score  N (%) | 17  24 (73) | 0  9 (27) | *0.065* | 0.0  29 (72) | 0.0  16 (28) | *0.730* |
| **Financial difficulties**  Median score  N (%) | 0  23 (74) | 0  8 (26) | *0.907* | 0.0  29 (72) | 0.0  11 (28) | *0.714* |

*Non-parametric test for continuous variables. For functional scores, a high score indicates a high functional level, for symptom scores a high value indicates an increased severity of symptoms.

*Non-parametric test for continuous variables. For functional scores, a high score indicates a high functional level, for symptom scores a high value indicates an increased severity of symptoms.
